# Drug Repurposing of New Treatments for Neuroendocrine Tumors

**DOI:** 10.3390/cancers17152488

**Published:** 2025-07-28

**Authors:** Stefania Bellino, Daniela Lucente, Anna La Salvia

**Affiliations:** National Center for Drug Research and Evaluation, Istituto Superiore di Sanità, 00161 Rome, Italy; daniela.lucente@iss.it (D.L.); anna.lasalvia@iss.it (A.L.S.)

**Keywords:** drug repurposing, drug cancer, neuroendocrine tumors, clinical perspectives

## Abstract

Drug repurposing or drug repositioning is the process of identifying new therapeutic uses for approved or investigational drugs beyond the original treatment indication. The discovery of new drugs for cancer therapy needs this cost-effective and time-saving alternative strategy to traditional drug development for a rapid clinical translation in Phase II/III studies, especially for unmet medical needs and rare diseases. Neuroendocrine tumors (NETs) are a heterogeneous group of rare neoplasms arising from cells of the neuroendocrine system that, though often indolent, can be aggressive and metastatic. In this context, drug repurposing has emerged as a promising strategy to improve treatment options due to the limited number of effective treatments and the heterogeneity of the disease. Indeed, a large number of non-oncology drugs have the potential to address more than one target that could be therapeutic for cancer patients. Although many repurposed drugs are used off-label, efficacy for the new use must be demonstrated in clinical trials. Within regulatory frameworks, both the Food and Drug Administration (FDA) and the European Medicines Agency (EMA) have procedures to reduce the need for extensive new studies and to expedite the review of drugs for serious conditions when preliminary evidence indicates substantial clinical improvement over available therapy. In spite of several advantages, including reduced development time, lower costs, known safety profiles, and faster regulatory approval, difficulty in obtaining new patents for old drugs with limited protection for intellectual property may reduce commercial returns and disincentivize investments. This review aims to provide comprehensive information on some marketed drugs currently under investigation to be repurposed or used in clinical practice for NETs and to discuss the major clinical challenges. Although drug repurposing is a useful strategy for early access to medicines, the monitoring of the clinical benefit of oncologic drugs during the post-marketing authorization is crucial to support the safety and effectiveness of treatments.

## Simple Summary

The identification of new therapeutic uses for drugs that are already approved for other diseases, known as drug repurposing, represents a challenging yet promising area of research. This concept has gained significant attention, particularly in the oncology field. The present work aims to provide a comprehensive overview of drugs that have been investigated as potential treatments for neuroendocrine tumors (NETs) as repurposed agents. While only a few compounds showed clinical efficacy in this area, everolimus stands out as a key example, and this immunosuppressant agent has become a cornerstone in the treatment of NETs. Other medications, such as metformin and statins, also have the potential to be included in the therapeutic options for these tumors, although conclusive evidence for their efficacy is lacking. Drug repurposing is considered a valuable strategy for accelerating drug development and ultimately improving the management of patients with NETs.

## 1. Introduction

Drug repurposing for cancers is a strategy of using existing drugs originally developed for other indications to treat various forms of tumors [1]. This approach leverages known pharmacological and safety profiles, potentially saving time and cost in drug development. Continued research is essential for identifying previously overlooked targets and mechanisms, as well as for anticipating and mitigating potential risks. Advancements in genomic and proteomic technologies, multi-omics approaches, single-cell sequencing, and the expansion of artificial intelligence provide opportunities for drug repurposing.

Neuroendocrine tumors (NETs) are a heterogeneous group of rare neoplasms arising in any site of the body from cells of the diffuse endocrine system, although they most frequently have a gastro-entero-pancreatic (GEP) origin (specifically, in about 60% of cases) and pulmonary in approximately 30% of cases [2]. The incidence of these neoplasms has progressively increased over the last few decades, reaching 6.98 new cases per 100,000 population (with a 6.4-fold increase from 1973 to 2012). NETs are typically categorized based on their morphology and proliferation index (mitotic count or Ki-67 index through immunohistochemistry). The latest World Health Organization (WHO) 2022 pathological classification of neuroendocrine and endocrine tumors classifies NETs as grade 1, grade 2, or grade 3, which are associated with progressively increasing proliferation rates and, therefore, variegated clinical behavior [3]. On the other hand, poorly differentiated neuroendocrine carcinomas (NECs) are highly aggressive neoplasms with a dismal prognosis.

NETs can produce hormonally active substances, which may lead to unique syndromes such as carcinoid syndrome, caused by the excessive secretion of serotonin. These tumors can arise in various primary sites throughout the body, including the ovary, kidney, or sinonasal tract, with significant variability in their biological aggressiveness, ranging from well-differentiated low-grade tumors to rapidly proliferating neuroendocrine carcinomas. Additionally, their clinical presentation can differ widely, e.g., functioning tumors may present symptoms typical of hormonal syndromes, such as diarrhea and flushing in carcinoid syndrome. This diversity creates specific challenges for diagnosis and clinical management [4,5].

For localized disease, curative radical surgery represents the standard of care, while for locally advanced unresectable or metastatic NETs, many systemic options are approved and available, including somatostatin analogues (SSAs), targeted therapies such as everolimus or sunitinib, peptide receptor radionuclide therapy (PRRT), and chemotherapy (e.g., temozolomide-based or streptozotocin-based) [5,6].

Predictive biomarkers to select the best therapeutic option for patients with NETs have traditionally been represented by the widespread presence of somatostatin receptors (SSTR), a target that has proven invaluable for diagnosis and therapy (i.e., SSAs and PRRT). However, validated predictive biomarkers for targeted agents or chemotherapy regimens are still lacking and not available for clinical use, despite considerable efforts to identify them. In this context, many potential predictors of mTOR inhibitor response have been proposed, but efforts to standardize these biomarkers have largely failed, presumably due to the complexity of the PI3K/mTOR pathway, which contains multiple feedback loops that could have unforeseen consequences. Furthermore, it is very difficult to standardize immunohistochemistry and molecular techniques, and while genomic and epigenetic approaches show promise, they require precise validation before being used in clinical settings [7]. Therefore, despite the identification of SSTR expression on the cell surface as a valuable predictive tool and therapeutic target, the treatment choices beyond SSA-targeted therapies currently accessible remain quite restricted, and ultimately, all patients build resistance to these medications. Significant efforts are deemed necessary to address resistance and create innovative strategies that enhance the treatment benefit–risk ratio for these patients, which includes discovering new therapy targets and identifying biomarkers that enable better selection of patients for tailored care.

With these premises, the clinical need to improve patients’ outcomes (above all in patients with advanced disease, and, as well in selected settings as in NET G3, which is a recently introduced category with particularly challenging management due to limited data to inform treatment strategies) together with a constant effort to ameliorate patients’ quality of life, reducing the side effects of the approved therapies and saving costs for the national health system, has paved the way for the repurposing of non-antineoplastic drugs as a potentially effective treatment for NETs. Many of these repurposed drugs act by mTOR inhibition, angiogenesis suppression, or cell cycle interference, targeting common pathways in NET biology.

Within regulatory frameworks, the Food and Drug Administration (FDA) 505(b)(2) New Drug Application (NDA) pathway allows pharmaceutical companies to seek approval for new uses of previously approved drugs relying on some existing data to reduce the need for extensive new studies [8]; in addition, the Breakthrough Therapy designation is a process to expedite the review of drugs for serious conditions when preliminary evidence indicates substantial clinical improvement over available therapy [9]. At the same time, the European Medicines Agency (EMA) has the Type II variation process when the change is not an extension of the marketing authorization (line extension) and may have a significant impact on the quality, safety, or efficacy of a medicinal product [10]. Real-world implementation must be planned early, especially from a Health Technology Assessment (HTA) perspective. The way treatments are delivered in clinical trials often differs significantly from routine practice. Anticipating these differences and planning for them can help avoid barriers to access and integration into healthcare systems.

## 2. Techniques for Drug Repurposing

Drug repurposing entails selecting a drug, assessing its efficacy using preclinical models, and advancing to pivotal clinical trials. Drug candidate identification can be achieved through both computational and experimental methods [11].

Computational repurposing techniques analyze existing data using sophisticated analytical methods to discover new possible drug–disease connections [12]. The molecular approaches are based on understanding drug activity and disease pathophysiology; they are often powered by large-scale molecular data known as omic data, including genomic, transcriptomic, or proteomic data and data based on drug targets and chemical structure. The real-world data approach focuses on identifying relationships between drugs and diseases and includes network-based, ligand-based, and structure-based drug repurposing and machine-learning techniques. Network-based computational biology integrates the relationship between biological molecules into networks to discover new properties at the network level and investigate how cellular systems induce different biological phenotypes under other conditions. Ligand-based approaches are evaluated because similar compounds have similar biological properties. These methods have been widely used in drug repurposing to analyze and predict the activity of ligands for new targets [13]. The widespread use of computational pharmacology has been facilitated by the availability of extensive data from various sources, such as genomics, proteomics, chemo-proteomics, and phenomics. This has led to the accumulation of not only data that characterizes disease phenotypes and drug profiles but also complete pathway maps. Furthermore, the development of next-generation computational methods using Artificial Intelligence (AI) and Machine Learning (ML) has positively impacted the different stages of drug development, playing an essential role in silico chemogenomics [14].

Experimental repurposing approaches include various laboratory and clinical methods such as binding assays (assessing the interaction between a drug and a specific biological target), phenotypic screening (testing a drug’s effect on a cellular or organismal level), in vitro/in vivo experiments, and clinical studies [15].

Finally, hybrid approaches, combining computational prediction with experimental validation, can be particularly useful for complex diseases like cancer, where multiple disease mechanisms are involved. Successful drug repurposing efforts typically involve a combination of computational predictions, experimental validation, and ultimately, clinical trials. For instance, a computational approach might identify candidate drugs, which are then screened in vitro and in vivo, and finally evaluated in clinical trials for new indications.

## 3. Drug Repurposing for Neuroendocrine Tumors (NETs)

### 3.1. Significance and a Few Successful Examples

Well-differentiated NETs are often associated with a relatively good prognosis, thanks to their indolent clinical behavior, thus representing a potentially ideal field for drug repurposing, allowing time to test repurposed agents. In this context, key molecular targets in the NETs field may align with known drug mechanisms. In addition, existing safety profiles of repurposed drugs could reduce risk and speed up clinical trials. Several marketed drugs are being explored or showing promise for the treatment of NETs, but only a small percentage have fulfilled a clinical unmet need and entered clinical practice.

**Figure 1:**
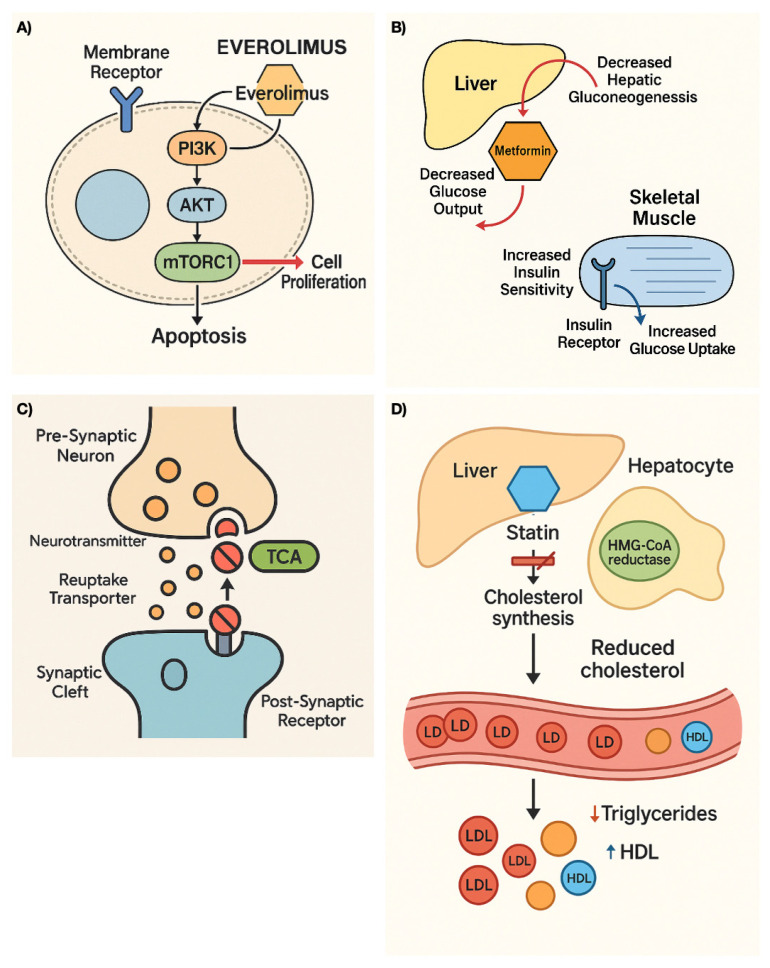
Mechanism of action of some repurposing drugs for NETs. (**A**) Everolimus, (**B**) Metformin, (**C**) Tricyclic antidepressants, (**D**) Statins.

Drugs currently being considered for repurposing in NETs, along with their mechanisms of action, clinical indication with level of evidence, and repurposing approach, are summarized in Table 1. In addition, Figure 1 illustrates the mechanism of action of some drugs.

Currently, everolimus (Afinitor^TM^, Novartis Europharm Limited, Dublin, Ireland) is the only marketed drug, developed as an immunosuppressant for the prevention of organ rejection in kidney and heart transplant recipients, which was repurposed for NETs based on the RADIANT trials demonstrating improved patient outcomes [16]. In these phase II and III studies, indeed, everolimus was associated with a significant increase in progression-free survival (PFS) in NETs with different primary origins. Stomatitis, diarrhea, fatigue, infections, rash, and peripheral oedema were among the most common adverse events (AEs) associated with everolimus, and they were typically of low grade (grade 1 or 2). Therefore, the anticancer drug everolimus has been shown to be associated with a manageable safety profile; the majority of everolimus-related AEs are controllable by stopping or modifying the dosage. Everolimus is an orally administered analog of rapamycin that acts as a molecular inhibitor of the mammalian target of rapamycin (mTOR) signaling pathway, and its mechanism of action involves the disruption of mTOR signaling through its high-affinity binding to the cytosolic protein FKBP-12. This interaction attenuates mTOR-mediated regulation of key cellular processes, including cell growth, proliferation, and angiogenesis [17]. The evidence of everolimus activity emerged across a broad spectrum of NETs, including those arising from the pancreas, lung, and gastrointestinal tract [18,19]. Since the initial approval by both FDA and EMA in 2011, important changes in the therapeutic/diagnostic and classification of NETs have occurred. Randomized phase III studies assessing SSAs, targeted therapies such as everolimus and sunitinib, and PRRT have shaped the present treatment guidelines. Unfortunately, the reduced number of head-to-head studies has hindered the establishment of the best treatment sequence. An effort has been made with the academic SEQTOR trial, which was aimed at evaluating the best therapeutic sequence in pancreatic NETs. This randomized open-label phase III study compared the efficacy and safety of everolimus followed by chemotherapy with streptozotocin and 5-fluorouracil or the reverse sequence [20]. PFS did not significantly differ between the two sequential strategies. However, when tumor shrinking is a priority, streptozotocin-based chemotherapy has emerged as the first preferred treatment. The significantly greater overall response rate (ORR) of everolimus observed in this trial supports this claim. In clinical practice, the therapeutic strategy is generally performed by a multidisciplinary team of NET specialists, including oncologists, endocrinologists, nuclear medicine physicians, radiologists, pathologists, radiotherapists, and surgeons. Specifically, the approach should be tailored to the individual circumstances of each patient, including tumor type and primary origins, tumor grade, somatostatin receptor (SSTR) expression (evaluated through functional imaging with 68-Gallium PET-CT), disease evolution, and patient-related factors like age, performance status, symptoms related to the disease, and personal preferences. Taking into account all these aspects, everolimus remains a key therapeutic agent in the treatment of advanced NETs, which covers the spectrum of this heterogeneous group of neoplasms, even if clear predictive biomarkers for its use are still lacking [21]. Nevertheless, challenges such as mechanisms of resistance, toxicity, and optimal therapeutic sequence remain unresolved [22].

### 3.2. Drugs with Clinical Significance and a Potential Role in the Field of NETs

The identification of new therapies with low toxicity and good tolerability, in particular for the treatment of pancreatic neuroendocrine tumors (pNETs), is a valuable goal to be pursued. In this perspective, metformin, a widely used agent for the treatment of patients with type 2 diabetes mellitus (T2DM), is emerging as a molecule of interest. The adenosine monophosphate-activated protein kinase (AMPK)-mediated inhibition of mTOR, along with other AMPK-dependent and independent mechanisms, contributes to metformin’s ability to reduce cancer cell proliferation, induce apoptosis, and interfere with tumor growth [23]. A systematic review evaluated the role of T2DM and metformin in the insurgence and post-treatment outcomes in patients with pNET [24]. In addition, an Italian multicenter retrospective study, including unresectable well-differentiated pNETs, demonstrated that metformin was associated with the increased PFS of patients receiving somatostatin analogues and/or everolimus [25]. However, the lack of prospective studies limits the possibility of exploring the therapeutic effect of metformin for pNETs.

Statins, which are primarily used to treat hypercholesterolemia, have demonstrated potential as anti-tumor agents and may enhance the effects of other therapies, even if conclusive evidence is missing. Through competitive HMG-CoA inhibition, statins reduce mevalonate synthesis, farnesylation, and geranylation, causing a decrease in hematic cholesterol levels. At the same time, they can reduce the activity of proteins associated with tumor proliferation, metastasis, and neo-angiogenesis, also influencing cell apoptosis by activating several caspases [26]. Lipid-lowering agents, including statins, have been explored as new therapies and useful tools in cancer prevention and tumor-growth control, but the absence of data coming from randomized clinical trials prevents drawing conclusions [27]. A recent observational multicenter retrospective study showed that statin therapy was associated with improved PFS among dyslipidemic NET patients, suggesting a potential antiproliferative effect of statins [28].

Both metformin and statins are widely used medications that could be valuable for repurposing in patients with NETs due to their mechanisms of action and potential clinical benefits. Retrospective studies have indicated that these primarily non-oncological agents may provide a survival advantage for NET patients [25,26,27,28]. However, further prospective studies are necessary to confirm the role of statins in managing NETs.

### 3.3. Drugs with Preliminary Clinical Data in the Field of NETs

A systematic drug repositioning bioinformatic approach, querying a large collection of gene expression profiles, was used to identify candidate-approved drugs to treat small cell lung cancer (SCLC). Tricyclic antidepressants (TCAs) were found to induce apoptosis in both chemonaïve and chemoresistant SCLC cells in culture and in “in vivo” models. In the same work, the authors showed that the candidate drugs inhibited the growth of other types of neuroendocrine neoplasms, including pNETs and Merkel cell carcinoma. Taken together, these data could help in the identification of novel targeted strategies for these tumors [29]. The therapeutic potential of TCAs in treating SCLC and other NETs is further elucidated by examining their mechanisms of action, primarily through the activation of stress pathways and the induction of cell death. This process is partly mediated by the disruption of autocrine survival signals involving neurotransmitters and their G protein-coupled receptors (GPCRs). The mechanisms by which TCAs such as imipramine and clomipramine exert these effects include the inhibition of serotonin and epinephrine reuptake, as well as antagonism of various receptors like cholinergic, histaminic, and adrenergic receptors. A bioinformatic approach carried out in preclinical models revealed a potential new use of imipramine in SCLC and other high-grade NETs [30]. Based on promising preclinical evidence, a phase IIa dose escalation study with desipramine was conducted on six patients: three with SCLC and three with non-SCLC NETs. However, the tolerability of desipramine was poor, and no clinical benefit was observed [31].

Thalidomide, an oral agent with antiangiogenic and immunomodulatory properties, has been investigated extensively in the management of advanced cancer. The mechanism of action is complex, and it probably includes different molecular targets. Thalidomide inhibits angiogenesis by interrupting processes mediated by bFGF and/or vascular endothelial growth factor (VEGF) and prevents TNF-α synthesis by inducing TNF-α mRNA degradation. Recent data suggest that this drug can also block the activation of nuclear factor (NF)-κB through a mechanism involving the inhibition of IκB kinase activity [32,33]. Currently, it is indicated (in combination with melphalan and prednisone) as a first-line treatment for patients with untreated multiple myeloma aged ≥65 years or for those who are ineligible for high-dose chemotherapy [34]. Those studied as a single agent showed no responses in NET patients. However, the combination with temozolomide resulted in a 25% radiological response rate, particularly in pNETs. Despite this, the combination caused significant toxicity, leading to early discontinuation in many patients [35]. It should be pointed out that temozolomide has a well-known activity in NETs, and the add-on value of thalidomide is still far from being elucidated with the available data [36]. Thereby, although early-phase studies indicated the potential efficacy of thalidomide in combination with temozolomide, further phase II studies investigating the combination in advanced/metastatic pancreatic and non-pancreatic NETs showed that S-1/temozolomide with or without thalidomide led to a comparable treatment response [37].

### 3.4. Drugs with a Preclinical Rationale Without Clinically Proven Implications

Pituitary neuroendocrine tumors (PitNETs) usually require complex management, and a relevant number of patients do not respond to currently available pharmacological treatments, i.e., SSAs or dopamine-agonists. Thus, novel chimeric somatostatin/dopamine compounds (dopastatins) that could improve the medical treatment of PitNETs have been designed due to their enhanced efficacy in suppressing GH hypersecretion. Studies in vitro suggested that dopastatins could be an efficacious therapeutic option to be considered in the treatment of PitNETs [38]. While dopastatin showed promise in preclinical studies, further clinical research is necessary to fully assess its efficacy and safety in treating PitNET, and currently, dopastatin is under investigation in a phase II clinical trial (NCT04335357).

Disulfiram, traditionally used to treat chronic alcoholism, has recently gained attention for its potential anticancer properties. Preclinical studies showed that disulfiram appears promising as an anti-tumor agent for the treatment of PitNETs, decreasing the cell viability in vitro and in vivo and inducing coproptosis in pituitary tumor cells [39]. A previous study showed that the lethal mechanism of cuproptosis involves the disruption of specific mitochondrial metabolic enzymes in the mitochondrial tricarboxylic acid (TCA) cycle, leading to proteotoxic stress and cell death. Therefore, targeting cuproptosis drugs provides a new perspective for the treatment of tumors [40,41,42].

Although chloroquine/hydroxychloroquine are known as antimalarial agents, scientific evidence also supports their use in the treatment of tumors, especially in combination with conventional anti-cancer treatments, potentiating therapeutic activity [43]. Chloroquine (CQ) and hydroxychloroquine (HCQ) exert anti-cancer effects through several key mechanisms. Primarily, they inhibit autophagy by disrupting lysosomal acidification, a critical process for the degradation and recycling of cellular components; additionally, these drugs can modulate signaling pathways involved in cancer progression. CQ has been shown to inhibit the TLR9/nuclear factor kappa B signaling pathway, thereby reducing cancer invasiveness, and both CQ and HCQ can suppress cancer cell proliferation by interfering with the CXCL12/CXCR4 signaling pathway. Furthermore, CQ can influence the p53 pathway by stabilizing the p53 protein and activating the transcription of pro-apoptotic genes. These multifaceted actions contribute to the potential of CQ and HCQ as anti-cancer agents, particularly in combination therapies. Preclinical studies suggested that autophagy inhibition by chloroquine/hydroxychloroquine could be used for the treatment of pNETs, including the well-differentiated type [44]. While these findings are favourable, the use of chloroquine/hydroxychloroquine in NETs remains investigational, and further studies are necessary to assess efficacy and monitor potential side effects.

Among nonsteroidal anti-inflammatory drugs (NSAIDs), celecoxib is considered particularly promising as an antitumor drug because of both selective COX-2 inhibition and powerful COX-independent toxicity on tumor cells. PitNET tissue has been identified to have high expression levels of COX-1 and COX-2, theoretically providing potential targets for NSAIDs. However, research is still in the early stages, and further investigation is needed to explore the molecular mechanisms and signal transduction pathways of NSAIDs in PitNET progression [45].

Neuroendocrine prostate cancer (NEPC) represents a highly aggressive subtype of prostate tumors. Levetiracetam, an antiepileptic drug, proved a potential effect in restraining neuroendocrine prostate cancer and inhibiting the progression of prostate adenocarcinoma, particularly after androgen deprivation therapy [46]. The protein target of levetiracetam, SV2A, is highly expressed by both NEPC cells and mast cells infiltrating prostate adenocarcinoma, while it is low or negligible in adenocarcinoma cells. In vitro, levetiracetam inhibited NEPC cell proliferation and mast cell degranulation.

Adopting a drug-repurposing strategy, fludarabine phosphate, used in the treatment of leukemia and lymphoma, was identified to inhibit the proliferation of N-MYC overexpressing NEPC cells by inducing reactive oxygen species (ROS) [47]. Therefore, the results indicate that increasing ROS production by the administration of fludarabine phosphate may represent an effective treatment option for patients with N-MYC-overexpressing NEPC tumors.

Finally, ketotifen, a medication used to treat mild asthma and allergic reactions in the eye, was identified as a potential therapeutic candidate for NEPC by drug screening utilizing an FDA-approved drug library [48]. In vitro experiments demonstrated that ketotifen effectively suppressed neuroendocrine differentiation, reduced cell viability, and reversed the lineage switch by targeting the IL-6/STAT3 pathway. The in vivo results showed that ketotifen significantly prolonged overall survival and reduced the risk of distant metastases in the NEPC mouse model, showing a potential therapeutic strategy for clinical application.

## 4. Conclusions

Drug repurposing is a strategy in drug discovery that aims to identify new uses for existing medications that have already been approved for other conditions. This promising approach can significantly reduce the time and cost associated with drug development by utilizing existing knowledge about the drug’s safety and pharmacological properties. As a result, it can shorten the overall timeline for bringing a drug to market and is particularly useful for addressing unmet medical needs and rare diseases, such as NETs. While only a few compounds showed clinical efficacy in this area, everolimus, an immunosuppressant agent, has become a key therapeutic agent for NETs. Other medications, such as metformin (used to treat Type 2 diabetes) and statins (primarily prescribed for hypercholesterolemia), may also serve as potential therapeutic options for these tumors; however, conclusive evidence for their efficacy has not yet emerged.

Novel targeted strategies with preliminary clinical data are being developed. Tricyclic antidepressants may be useful in treating SCLC. Additionally, dopastatin (used to reduce hormone production, especially in tumors), disulfiram (used for chronic alcoholism), and NSAIDs such as celecoxib might be effective treatment options for PitNETs. Chloroquine and hydroxychloroquine (antimalarial agents) could be used for pNETs, while levetiracetam (an antiepileptic drug) and fludarabine phosphate (used for chronic lymphocytic leukemia) may represent potential therapeutic candidates for NEPC, along with ketotifen (a medication used to treat mild asthma and allergic reactions).

The clinical data available with tentative or successfully repurposed drugs for NETs have several limitations, mainly regarding potential bias due to randomization, missing data, and the selection of reported results. To improve the scientific rigor and interpretation of the discussed results, a risk-of-bias analysis of the main clinical studies regarding drugs successfully repurposed for NETs (everolimus), drugs with a potential role in the field of NETs (metformin and statins), and drugs with preliminary clinical data (tricyclic antidepressants and thalidomide) has been performed and provided as Supplementary Materials.

Drug repurposing is gaining traction as a cost-effective and time-efficient alternative to developing new drugs. Looking ahead, the future of drug repurposing will likely be shaped by the integration of AI, precision medicine, and systems biology. AI and ML are transforming the field of drug repurposing by facilitating large-scale pattern recognition across heterogeneous datasets. Algorithms that analyze chemical structures, transcriptomic profiles, adverse event data, and clinical outcomes can now predict new drug–disease interactions with remarkable accuracy. As drug repurposing initiatives grow, the importance of precise biomarkers becomes increasingly vital. Most current approaches to repurposing depend on general disease-level associations; however, individual differences in drug response necessitate a more personalized strategy. Biomarkers, such as genetic mutations and transcriptomic signatures, can help categorize patient populations to identify those who are most likely to benefit from a repurposed drug. Future drug repurposing efforts will increasingly involve the integration of multiple omics fields, including genomics, transcriptomics, proteomics, metabolomics, and epigenomics.

Although drug repurposing offers a valuable strategy for gaining early access to new medicines, it is crucial to monitor the clinical benefits of oncologic drugs following their post-marketing authorization. This monitoring supports the ongoing assessment of the safety and effectiveness of these treatments.

## Figures and Tables

**Table 1:** Drugs currently being considered for repurposing in NETs.

Active Substance	Mechanism of Action	Clinical Indication (Level of Evidence)	Repurposing Approach
**Drug repurposed for NETs**			
Everolimus	mTOR inhibitor	pNETs extra pNETs (Phase III randomized controlled studies)	Experimental
**Drugs with clinical significance and potential role in the field of NETs**			
Metformin	Inhibits mitochondrial oxidative phosphorylation, leading to activation of AMPK and subsequent downregulation of the mTOR pathway.	pNET (Systematic review, Retrospective studies)	Experimental
Statins	Inhibit the enzyme HMG-CoA reductase in the liver, decreasing hematic cholesterol levels; reduce proteins involved in tumor proliferation and angiogenesis; induce cell apoptosis.	pNETs PitNETs SCLC Lung NETs Small Bowel NETs Pheochromocytoma Merkel cell carcinoma (Observational retrospective studies)	Experimental
**Drugs with preliminary clinical data in the field of NETs**			
Tricyclic Antidepressants	Inhibits serotonin and epinephrine reuptake, and antagonize cholinergic, histaminic, and adrenergic receptors. Activate stress pathways and in-duce cell death.	SCLC pNETs (Preclinical models, Phase II study)	Computational Experimental
Thalidomide	Inhibits angiogenesis by interrupting processes mediated by bFGF and/or VEGF. Inhibits TNF-α synthesis and blocks the activation of nuclear factor (NF)-κB.	pNETs (Phase II studies)	Experimental
**Drugs with preclinical rationale without clinically proven implications**			
Dopastatins	Enhanced efficacy in suppressing GH hypersecretion.	PitNETs (Preclinical studies)	Experimental
Disulfiram	Inhibits the enzyme aldehyde dehydrogenase (ALDH). Induces cuproptosis leading to proteotoxic stress and cell death.	PitNETs (Preclinical studies)	Experimental
Chloroquine Hydroxychloroquine	Inhibit autophagy by disrupting lysosomal acidification and can suppress cancer cell proliferation by interfering with the CXCL12/CXCR4 signaling pathway.	pNETs (Preclinical studies)	Experimental
Celecoxib	Inhibits cyclooxygenase-2 (COX-2), an enzyme involved in the production of prostaglandins, which are mediators of pain and inflammation.	PitNETs (In vitro studies)	Experimental (target mechanism-based)
Levetiracetam	Modulation of neurotransmitter release through binding to the synaptic vesicle glycoprotein 2A.	NEPC (In vitro experiments, preclinical studies)	Computational Experimental
Fludarabine Phosphate	Inhibits DNA synthesis, primarily by interfering with the activity of several enzymes involved in DNA replication.	NEPC (In vitro experiments)	Computational
Ketotifen	Suppresses neuroendocrine differentiation, reduces cell viability, and reverses lineage switch via targeting the IL-6/STAT3 pathway.	NEPC (In vitro experiments)	Computational

Abbreviations: AMPK, Adenosine Monophosphate-Activated Protein Kinase; pNET, pancreatic Neuroendocrine Tumor; PitNETs, Pituitary Neuroendocrine Tumors; SCLC, Small Cell Lung Cancer; NEPC, Neuroendocrine Prostate Cancer. Tricyclic Antidepressants (TCAs, e.g., Imipramine, Clomipramine, Desipramine); Dopastatins (Chimeric Somatostatin/Dopamine Compounds); Chloroquine (CQ)/Hydroxychloroquine (HCQ). All drugs are administered orally.

## Supplementary Materials

The following supporting information can be downloaded at https://www.mdpi.com/article/10.3390/cancers17152488/s1.
