# Supplementary material for: Drug Repurposing of New Treatments for Neuroendocrine Tumors
Source: Cancers (Basel). 2025 Jul 28;17(15):2488. doi: 10.3390/cancers17152488 (PMC12345725; doi:10.3390/cancers17152488)
Supplement: Supplementary file 1 [file cancers-17-02488-s001.zip › cancers-3745253-supplementary.pdf]

Supplementary Materials

Risk-of-bias assessment of the main clinical studies with the tentative and successfully repurposed drugs for NETs

Study/Domain	D1	D2	D3	D4	D5
1-Everolimus	+	+	+	+	+
2-Metformin	X	−	−	+	−
3-Statins	X	−	−	+	−
4-Tricyclic Antidepressants	−	+	+	+	+
5-Thalidomide	+	+	+	+	+

Domains: D1 bias due to randomization, D2 bias due to deviations from intended intervention, D3 bias due to missing data, D4 bias due to outcome measurement, D5 bias due to selection of reported results.
